# Culturally adapted meta-debriefing: a mixed-methods study of reflective practice in Spanish-speaking simulation faculty development in Chile

**DOI:** 10.1186/s41077-025-00380-0

**Published:** 2025-10-24

**Authors:** Soledad Armijo-Rivera, Karen Vergara, Scarlett Vicencio-Clarke, Brynjar Foss, Marcia Maldonado Holtheuer

**Affiliations:** 1 Facultad de Medicina Clínica Alemana, Universidad del Desarrollo, Santiago, Chile; 2 Universidad de Barcelona, Barcelona, Spain; 3 Laerdal Medical Corporation Latin America, Alphaville, Brazil; 4 Unidad de Simulación E Innovación en Salud, Universidad San Sebastián, Santiago, Chile; 5 Laerdal Medical Corporation, Stavanger, Norway; 6 Centro de Simulación, Innovación y Formación Interdisciplinar, Facultad de Salud y Odontología, Universidad Diego Portales, Santiago, Chile

**Keywords:** Debriefing, Meta-debriefing, Debrief the debrief, CORE debriefing, Simulation, Faculty development, Latin America

## Abstract

**Background:**

Simulation-based education has expanded across Latin America, creating demand for contextually relevant faculty development. Meta-debriefing, defined as a facilitated conversation following a debriefing, can strengthen debriefer performance. However, its implementation and effectiveness in Spanish-speaking settings remain underexplored. The CORE model (context, observation, reflection, and enhanced practice) provides structural guidance, but its adaptation to regional cultures has not been studied.

**Methods:**

We conducted a concurrent mixed-methods study of one experienced meta-debriefer interacting and facilitating 15 remote meta-debriefing sessions with interprofessional novice debriefers. Quantitative data were derived from audio-recorded sessions using DASH, SET-M, a CORE-aligned checklist, and a meta-debriefing pillars tool. Qualitative data consisted of written observer reports, analyzed using inductive content analysis, and triangulated across sources.

**Results:**

Quantitative analysis revealed high adherence to the CORE model and consistent demonstration of the four meta-debriefing pillars. Peer-assessed DASH scores ranged from 6.8 to 7.0, while SET-M items were consistently marked as achieved. Qualitatively, the meta-debriefer demonstrated behaviors aligned with psychologically safe, context-dependent, theoretically grounded, and formative facilitation. The meta-debriefer used cultural metaphors (e.g., “boli,” “cazuelas,” and a prisoner’s song) to clarify structure and normalize reflection. Five reflection strategies emerged as central to generating debriefer insight: (1) Structured reorientation, (2) constructivist linkage to clinical identity, (3) metaphorical framing, (4) strategic questioning (e.g., advocacy & inquiry, circular inquiry), and (5) emotional normalization.

**Conclusion:**

This study demonstrates how meta-debriefing, when implemented through culturally sensitive and structured approaches like CORE, supports transformative learning among simulation debriefers. Meta-debriefer’s relational style, use of shared narratives, and context-sensitive scaffolding activated learner-centered reflection. As meta-debriefing models gain traction in Latin America and beyond, this study highlights the value of culturally responsive faculty development strategies that integrate local language, values, and pedagogical traditions into simulation-based education.

## Background

Simulation-based education has become a cornerstone of modern healthcare training, providing a safe and controlled environment for learners to develop critical skills and enhance their decision-making abilities [1]. While the simulation itself provides a valuable learning opportunity, the debriefing process is crucial in enabling participants to reflect on their experiences, analyze their actions, and identify areas for growth [2].

Consequently, the quality of debriefing is a key determinant of simulation’s overall effectiveness, highlighting the critical need for targeted and ongoing faculty development in effective debriefing techniques [3] and the limited validity evidence of debriefing assessment tools [4].

Recent literature on the development of debriefing skills in simulation facilitators emphasizes the importance of structured approaches [5], the significance of context [6], continuous assessment [7], and the necessity of peer support to enhance debriefing quality [8]. Particularly, peer learning and mutual observation can promote a culture of professional improvement and support within simulation teams. These observations highlight the growing interest in meta-debriefing, a facilitated conversation that enables debriefers to reflect on and refine their practices [9].

More than just a post-session critique, meta-debriefing is a purposeful process designed to enhance debriefers’ self-awareness, refine their techniques, and enrich the skills of both novice and experienced debriefers. The article by Kumar et al. [9] presents a robust theoretical framework for effective meta-debriefing, grounded in four key pillars: theoretically driven (encompassing constructionist and constructivist theories), psychologically safe, context-dependent, and formative in function. Practical applications of this model, particularly in Spanish-speaking countries, have not been empirically studied.

The CORE debriefing model, developed during the pandemic to support debriefing training, introduces a four-phase structure: context, observation, reflection, and enhanced practice. Building on Steinwachs’ work [10], CORE emphasizes clarity of structure, alignment of learning outcomes, and actionable next steps. It is supported by an e-learning module and a flashcard-based cognitive aid to promote low-dose, high-frequency learning [11]. While CORE has been implemented in undergraduate midwifery training for student-led simulation [12], its use in meta-debriefing and how it is adapted or perceived in Latin American Spanish-speaking settings remain unexplored.

In Latin America, hierarchical dynamics and punitive responses to error significantly influence everyday practices within healthcare systems [13, 14] and asymmetric communication and abuse of power in residents’ training [15] and lack of emotional support in interns’ training [16]. In situ simulation (ISS) in a private ICU has been described as a powerful, emotionally engaging learning method [17]. Student reflections highlight how educators can reduce hierarchy, promote inclusion, adapt to diverse learning needs, and foster psychological safety [18]. Despite differences in study design and context, these findings show that organizational climate and learning approaches significantly influence speaking up, reflection, and system learning, ultimately shaping debriefing and meta-debriefing practices.

The goal of this study is to evaluate the CORE debriefing model on meta-debriefing practices in a Latin American Spanish-speaking context by (a) examining its process/structure and perceived effectiveness and (b) describing culturally resonant practices that arise within this approach through a mixed-methods approach.

In this study, the term “debriefer” refers to the faculty member leading the learner debriefing, the “meta-debriefer” to the faculty developer guiding the reflective session with debriefers, and the “researcher” to the study team involved in data collection and analysis.

## Methods

### Design

This study employed a concurrent mixed-methods research design [19] focused on the recorded interactions of 1 senior meta-debriefer during a series of 15 faculty development sessions in Chile [20]. The quantitative phase involves applying the DASH and SET-M to audio-recorded meta-debriefings provided by three researchers and as a self-assessment for the meta-debriefer. The qualitative phase consisted of analyzing written observations provided by the researchers, using inductive content analysis following the three-step approach described by Elo and Kyngäs [21]. This analysis focused on exploring the pillars of meta-debriefing and gaining deeper insight into the enhancement of practice learning points during meta-debriefings [9].

### Rationale for design

Given our aim to examine both the structure and perceived effectiveness of the CORE model, while also capturing culturally resonant practices, we selected a concurrent mixed-methods design. This approach allowed us to combine validated quantitative measures of debriefing behaviors and perceived effectiveness (DASH, SET-M) with context-specific checklists (CORE structure, meta-debriefing pillars) and a global rating. Together, these instruments supported triangulation across complementary constructs, enhancing internal validity. The qualitative strand (inductive content analysis) added depth by exploring how these processes unfolded in practice and revealed culturally embedded dimensions of meta-debriefing.

### Participants

The study included 1 experienced simulation physician as a meta-debriefer and 15 novice simulation debriefers, with no prior relationship or hierarchical connection between them. All of them were healthcare professionals from four cities in the same country, working at a single university institution. The experienced metadebriefer is a physician who has undergone several formal debriefing training courses, possesses over 20 years of experience leading simulation debriefings, and has 5 years of experience utilizing the CORE debriefing structure. The novice debriefers, who include nurses, physiotherapists, nutritionists, midwives, occupational therapists, speech therapists, and psychologists, have less than 1 year of experience facilitating debriefing after being approved in an institutional debriefing course, which was guided by two professionals who do not participate in the meta-debriefing practices or the analysis. The participation was voluntary. All debriefers participated in an informed consent process that began with the provision of detailed written instructions explaining the intervention. They also participated in a group remote session with the meta-debriefer to verbally explain the process and clarify the expected goals and content of the intervention. These actions functioned as a brief pre-meta-debrief discussion.

### Intervention

All novice debriefers were invited to participate in a remote immersive interprofessional simulation experience, where they guided a 20–30 min debriefing of medical and midwifery undergraduate students. By “remote simulation,” we mean synchronous, technology-mediated simulation activities where debriefers debriefed learners via videoconferencing platforms, rather than in person. The debriefers applied frameworks such as GAS, Plus-Delta, or CORE, depending on their individual preference and experience. The meta-debriefer observed the debriefing as a non-participant observer and, immediately afterward, conducted a one-on-one meta-debriefing using the CORE debriefing model. The meta-debriefings were guided on Zoom (Zoom Video Communications, Inc. (2023), Zoom cloud meetings (Version 5.7.1)), and audio recordings were downloaded.

### Instruments to gather quantitative data

To assess the meta-debriefing, we used the DASH short version [22, 23], the debriefing subscale of the SET-M [24, 25], and three purposefully developed instruments for this research.

a *DASH short version*: This instrument was included to assess facilitation behaviors during the meta-debriefing. The tool includes six items rated on a 7-point Likert scale, focusing on debriefer behaviors during the briefing and debriefing to evaluate engagement, structure, reflection, feedback, and performance improvement (Cronbach’s *α* > 0.8 in validation study) [22]. The instrument has a Spanish linguistically validated version, which was used in this study [23].
b *SET-M debriefing subscale*: This tool assessed perceived effectiveness in terms of learning and confidence, aligning with our objective of exploring meta-debriefing impact. The Spanish version of the Simulation Effectiveness Tool-Modified (SET-M) was employed [24]. This validated instrument comprises 19 items across 4 subscales: prebriefing, learning, confidence, and debriefing, with a Spanish version validated for use in remote simulations [25].
c *CORE checklist*: A 12-item dichotomous checklist was developed based on the actions described in the CORE flashcard (Table 1) to assess if the structure of the debriefing was followed. This allowed us to move beyond generic debriefing behaviors and evaluate adherence to the specific steps of the model.
d *Meta-debriefing pillars*: A five-item dichotomous checklist was developed based on the four meta-debriefing pillars, constructs not covered by existing instruments. The research team used the first pillar to generate the first two items: one for constructionist theories and the second for constructivist theories. The instrument developed includes an operational definition of each pillar (Table 1).
  Although the newly developed checklists (CORE and meta-debriefing pillars) have not yet undergone psychometric testing, their content was derived directly from CORE materials and operational definitions and refined by consensus within the research team, supporting strong face and content validity.
e *Global rating score*: A 1 to 7 global rating score debriefing performance was included to capture a global judgment of quality, recognizing this as a pragmatic measure commonly used in faculty development contexts.

**Table 1:** CORE debriefing actions and meta-debriefing pillars observed by peers (researchers) and self-assessment (meta-debriefer)

CORE structured actions	Peer assessment		Self-assessment	
	*n*	%	*n*	%
1. Expert opened by defining the context and expressed the time for the meta-debriefing	44	97.8	15	100
2. Expert opened by defining the context and recalled the learning objectives	44	97.8	15	100
3. The expert started defining the context and allowed to express the immediate reactions after the debriefing	45	100	15	100
4. The expert started the observation and asked for a synthesis of the facts	45	100	15	100
5. The expert opened the reflection by addressing the positive aspects of debriefing	45	100	15	100
6. The expert continued the reflection by addressing areas for improvement	45	100	15	100
7. The expert provided specific recommendations for improvement during the reflection addressing areas for improvement	45	100	15	100
8. The expert closed the debriefing by inviting participants to identify actions that would enrich the educator’s debriefing practice	45	100	15	100
CORE unstructured actions	Peer assessment		Self-assessment	
	*n*	%	*n*	%
9. The expert started the observation and when asking for the synthesis asked not to analyze	40	88.9	15	100
10. The expert stopped the observation in case the participant started to analyze	34	75.6	4	26.7
11. The expert stopped the reflection in case the participant started to analyze the negative aspects, emphasizing the importance of reinforcing the positive aspects first	30	66.7	5	33.3
12. The expert used “pearls” during the reflection addressing areas for improvement	41	91.1	15	100
Meta-debriefing pillars observed	Peer assessment		Self-assessment	
	*n*	%	*n*	%
The expert demonstrated that he/she can apply constructionist theories to guide the meta-debriefing (e.g., referred to societal or cultural influences, including when formulating conversation segments in which his/her own culture surfaces in the meta-debriefing). This means that he applies Bandura’s theory, vicarious learning, etc.	45	100	15	100
The expert demonstrated that he can apply constructivist theories to guide the meta-debriefing (e.g., he guided his facilitation for the educator to relate his previous experiences to the debriefing analyzed). This means that he applied experiential learning theories	45	100	15	100
The expert demonstrated skills in building a climate of psychological safety that allowed the educator to actively engage in the discussion and construction of learning conclusions in the meta-debriefing process	45	100	15	100
The expert conducted the meta-debriefing considering the professional, cultural, and institutional context in which the strategy was implemented, in order to maximize the educator’s reflection	45	100	15	100
The expert conducted the meta-debriefing with an eminently formative intention, aimed at supporting the educator’s professional growth and development toward adaptive expertise	45	100	15	100

### Procedure

The audio recordings of the meta-debriefing sessions were anonymized and transcribed verbatim by a single researcher. Subsequently, three simulation researchers and the meta-debriefer independently analyzed the anonymized recordings. All of them held a Master’s degree in Medical Education, had formal training in debriefing, had completed specific training in the CORE debriefing model, and had experience using DASH or SET-M. Each also had prior experience in quantitative research, and two of them had experience in qualitative content analysis. After listening to the recordings, each researcher completed the set of four instruments and the global rating scale to assess the quality of the meta-debriefings quantitatively.

Additionally, each researcher generated a written report, which was used for the qualitative analysis [21]. The researchers focused their observations on facilitation behaviors and reflective strategies aligned with the four core pillars of meta-debriefing: theoretically driven (encompassing constructionist and constructivist theories), psychologically safe, context dependent, and formative in function. The researchers also focused on the insights gained by participants into the “Enhancement of practice” step during the meta-debriefings. These conceptual domains guided their observations thematically. Each comment was explicitly linked to a specific performance, capturing formative and reflective insights intended to support the professional development of the novice debriefers. The nature of the data was brief yet purposeful, typically ranging from short phrases to one or two sentences (e.g., “validates emotion before moving on,” “reframes performance without judgment,” or “skips reflective questioning”).

### Quantitative analysis plan

Quantitative data were analyzed using descriptive statistics. For the CORE checklists, as well as for the meta-debriefing pillars, frequencies and percentages of affirmative responses were calculated based on peer and self-assessments. For the SET-M instrument [24, 25], the frequency and percentage of higher scores (3 points) were reported for each item, separately for peer (researchers) and self-assessments (meta-debriefer). For the DASH instrument [22, 23], item-level scores were summarized using the mean, standard deviation, minimum, and maximum values for both peer and self-assessments. The global rating score was reported separately using the mean for each assessment type. All analyses were conducted using JASP software (version 0.19.1).

### Qualitative inductive content analysis

After familiarizing themselves with the written reports, two members of the research team performed an inductive content analysis of the written reports [21]. Each text was coded and grouped under one or more of the four meta-debriefing pillars, guided by a combination of theoretical alignment and semantic proximity. Cultural markers, as phrases, metaphors, or references specific to the Latin American context, were identified as part of the constructionist and constructivist subthemes. To enhance the validity and integration of findings, two researchers implemented a triangulation process, following the guidelines for concurrent mixed-methods research described by Creswell and Plano Clark [19]. Methodological triangulation was applied by concurrently collecting quantitative and qualitative data. Data source triangulation was achieved through the inclusion of self and peer assessments, allowing for multiple perspectives on meta-debriefer performance. Investigator triangulation was ensured by engaging four trained raters for the quantitative phase and two independent coders for the qualitative analysis, each with formal training in simulation and qualitative methods. Analytical triangulation was conducted through a side-by-side comparison of results, examining the alignment and divergence between numeric scores and emergent themes related to the CORE framework and meta-debriefing pillars. Considering that the goal of meta-debriefing is to improve the competencies and reflexivity of the debriefer, the inclusion of the meta-debriefer as a researcher provides internal validity to the triangulation process. This integration process enabled the research team to generate meta-inferences that enriched the interpretation of facilitation strategies and instructional effectiveness in meta-debriefing contexts, summarized in the results report [26].

The project was approved by the Ethics Committee of Universidad San Sebastián (Act 169–23). All the participants were volunteers and gave informed consent.

## Results

To align with the concurrent mixed-methods design, the following section presents the quantitative analyses first, followed by a description of the qualitative triangulation process used to integrate data strands.

### Quantitative analysis

Fifteen meta-debriefings were evaluated. Structured CORE actions were consistently observed, with research assessments reporting 100% compliance in six of the eight items and 97.8% in the remaining two (opening by defining the context and available time and recalling learning objectives), while meta-debriefer self-assessments indicated full compliance across all items. Unstructured actions showed greater variability. Researchers noted that the meta-debriefer instructed debriefers to describe the case without analysis in 88.9% of sessions, actively redirected debriefers in 75.6%, and intervened when prematurely focused on negative aspects in 66.7%. These latter actions were less frequently reported in meta-debriefer self-assessments (26.7% and 33.3%, respectively). All five pillars of meta-debriefing were present in 100% of sessions according to both peer (*n* = 45) and self-assessments (*n* = 15) (Table 1).

In the SET-M debriefing subscale, five of the seven items were rated as fully achieved (100%) by researchers. The remaining two, “the instructor was able to verbalize their emotions before focusing on the facts” and “the debriefing provided an opportunity to reflect on their performance,” received agreement from 97.8% and 93.3% of researchers, respectively. All items were marked as achieved (100%) in the meta-debriefer self-assessment (Table 2).

**Table 2:** Peer (researchers) and self-assessment (meta-debriefer) of debriefing experience using SET-M debriefing section

SET-M debriefing section	Peer assessment higher scores		Self-assessment higher scores	
	*n*	%	*n*	%
1. The debriefing conducted by the expert contributed to the educator’s learning	45	100	15	100
2. The educator was able to verbalize their feelings before focusing on the facts of the debriefing discussed with the expert	44	97.8	15	100
3. The debriefing conducted by the expert was valuable in helping the educator improve their debriefing skills	45	100	15	100
4. The debriefing conducted by the expert gave the educator the opportunity to reflect on their performance during the debriefing	42	93.3	15	100
5. The debriefing conducted by the expert was a constructive evaluation of the educator’s debriefing	45	100	15	100
6. The expert managed to maintain a safe atmosphere in the debriefing	45	100	15	100
7. The expert helped the educator to reflect deeply on his or her own performance	38	84.4	15	100

In the DASH short version, researcher assessments yielded very high scores across all six items (6.80–7.00 on a 1–7 scale). The highest ratings (7.00, *SD* = 0.00) were for maintaining a participatory environment and structuring the debriefing, while the lowest (6.80) was for generating deeper reflective discussions. Meta-debriefer self-assessments were slightly lower (6.53–6.60) and more variable (*SD* = 0.74–0.83), with the largest difference in item 2 (7.00 vs. 6.60). Overall, both assessments reflected consistently high performance, with minimum scores of 4.00 (researchers) and 5.00 (meta-debriefer) and maximums of 7.00 across all items (Table 3).

**Table 3:** Peer (researchers) and self-assessment (meta-debriefer) of meta-debriefing experience using DASH short version

DASH		Mean	SD	Min	Max
1. The expert established an environment for a participatory learning experience (introduced him/herself, said what would be done, explained the weaknesses and strengths of the session, invited the participant to share his/her thoughts)	PA	6.90	0.47	4.00	7.00
	SA	6.60	0.74	5.00	7.00
2. The expert maintained a participatory learning environment (said what was expected of the educator, focused on making him/her learn and not on making him/her feel bad, allowed him/her to speak)	PA	7.00	0.00	7.00	7.00
	SA	6.60	0.83	5.00	7.00
3. The expert structured the debriefing in an organized way (the conversation was agile, at the beginning he invited to share reactions or emotions, in the middle there was analysis of the case, and at the end a synthesis or application phase of the session)	PA	7.00	0.00	7.00	7.00
	SA	6.60	0.83	5.00	7.00
4. The expert generated deep discussions that made the learner reflect on his performance (he gave concrete examples, his ideas were clear, he was respectful, and he helped him learn)	PA	6.80	0.46	5.00	7.00
	SA	6.53	0.74	5.00	7.00
5. The expert identified what the learner did well or poorly—and why (gave targeted feedback on performance and helped the learner identify important points in reflection)	PA	6.96	0.21	6.00	7.00
	SA	6.53	0.74	5.00	7.00
6. The expert helped the learner to see how to achieve or maintain good performance (helped the learner to learn and improve performance, and the expert has good level of knowledge and made sure to address important issues)	PA	6.84	0.42	5.00	7.00
	SA	6.60	0.74	5.00	7.00

*PA* peer assessment, *SA* self-assessment, *SD* standard deviation, *Min* minimum, *Max* maximum

### Qualitative analysis

An inductive content analysis process was employed to gain a deeper understanding of the meta-debriefing pillars and culturally resonant practices.

For transparency and to preserve cultural nuance, participant quotations are presented in both the original Spanish and their English translation.

#### Psychologically safe: signaling permission to feel and speak freely

Throughout the debriefing sessions, the meta-debriefer consistently created conditions of psychological safety by validating emotional responses, paraphrasing with care, and checking in before deepening reflection. According to the researchers, the behavior was not merely affective but purposeful; it opened cognitive space by first acknowledging emotional truth and is described using expressions as follows:

Facilitate the conversation, giving space to think./Facilitar la conversación, dando espacios para pensar

Normalize the response of how do you feel, thus transforming the feeling to start the conversation./Normaliza la respuesta del ¿cómo te sientes? de esta forma transforma el sentir para poder comenzar a conversar

Tone modulation and empathetic reframing were used not to soften the challenge but to make the debriefer feel seen. In several instances, the meta-debriefer redirected the conversation away from harsh self-criticism and toward a constructive interpretation, reinforcing the importance of safety as a prerequisite for reflection.

#### Context dependent: reading the room, the role, and the region

The meta-debriefer regularly adapted his facilitation to match the debriefer’s level of experience, role, and the institutional context. When a debriefer expressed discomfort with technical simulation aspects, the response, “it's perfect, nothing happens with that,” was more than reassurance; it was an acknowledgment of pedagogical diversity.

The meta-debriefer was careful to take the anxiety out of handling the technical aspect by understanding the diversity in debriefer training./El meta-debriefer se preocupó por quitar la ansiedad del manejo del aspecto técnico entendiendo la diversidad en la formación de los debriefers

The meta-debriefer frequently contextualized her observations by referencing typical student profiles, institutional pressures, or cultural expectations, demonstrating a deeply rooted understanding of the ecosystem in which reflection and change must occur.

#### Theoretically driven: constructing knowledge through dialogue and cultural metaphor

The meta-debriefer’s theoretical grounding was visible in both constructivist and constructionist modes. Constructivist practices included naming debriefing techniques (e.g., Plus-Delta, advocacy, and inquiry—A&I—questioning), correlating them to learning goals, and aligning them with experiential learning theory.

The application of constructivist theory through experiential learning was demonstrated by correlating techniques used in psychology…/Se demostró la aplicación de la teoría constructivista a través del aprendizaje experiencial al correlacionar técnicas que se usan en psicología…

Notably, the meta-debriefer employs a constructionist facilitation approach, richly embedded with cultural expressions and narrative strategies that resonate with Latin American identities. Rather than citing theory explicitly, the meta-debriefer often evoked shared meanings and metaphors to provoke insight. In one outstanding example, the meta-debriefer addressed a participant’s tendency to divert from the main point through a popular reference to a song by the band “Los Prisioneros” that refers to a 45° tangent, alluding to a person who drifts away from a goal, being swept away by a current of water.

She uses a ‘Los Prisioneros’ song to connect with what the debriefer said about going off on a tangent./Utiliza una canción de Los Prisioneros para conectar con lo expresado por la debriefer cuando habla de irse por la tangente.

After using this song reference, the debriefer laughed and affirmed the connection, illustrating how cultural metaphors can make abstract facilitation feedback more concrete, memorable, and shared. This example also highlights how metaphorical and colloquial language, when culturally meaningful, serves as a structural strategy that strengthens engagement and understanding during meta-debriefing.

Another example referenced a street game rule in Chile, used to ask for a pause, where players used verbally the expression “boli” in concomitance with a hand expression joining the thumb and index finger in a circle and holding the other fingers straight or relaxed away from the palm, to explain the use of structural strategies in briefing, a culturally grounded metaphor that blended playfulness with precision:

Explain what ‘boli’ or the basketball stop means to introduce the relevance of using some structures to optimise the briefing./Explica qué significa "boli" o el stop del básquetbol para introducir la relevancia de usar algunas estructuras para optimizar el briefing.

The hand gesture is used in other cultural contexts as a safety sign (Scuba diving), for meditation (Buddhism), or money (Japan), but when it is associated with the word “boli,” it means a totally different expression, culturally pertinent for young generations in Chile.

Such expressions made theoretical constructs more accessible, turning abstract concepts into shared, embodied knowledge. This blend of pedagogical depth and cultural fluency is particularly salient in Latin American educational contexts, where relational trust and metaphorical language are highly valued.

#### Formative in function: orienting the conversation toward growth

A defining feature of the meta-debriefer’s style was his consistent focus on development over correction. The facilitation was goal-oriented, yet never directive; the meta-debriefer invited the debriefer to reflect on what could be strengthened, not what was wrong. Recommendations were framed gently, often preceded by affirmations of what was already working.

Draws from reflection on strategies for improvement./Extrae desde la reflexión estrategias de mejora

The expert gave two specific recommendations for improvement./El experto entregó dos recomendaciones específicas para la mejora

Rather than imposing a model, the meta-debriefer helped the debriefer discover options, revealing how one’s style might evolve while staying authentic. This growth-focused stance aligns with both adult learning principles and regional pedagogical values, where mentorship and companionship are more persuasive than authority.

### Meta-debriefing perceived effectiveness

Drawing from paired observations of meta-debriefer self-reflection strategies and corresponding debriefer insights, we present thematic relationships that reveal how carefully crafted meta-debriefing moves can unlock meaningful debriefer development (Table 4).

**Table 4:** Relationships between meta-debriefer reflection strategies and debriefer insights

Meta-debriefer reflection strategies	Example	Debriefer insights	Example
Structured reorientation	Organize the participants’ diffuse ideas about what they did well and even list them	Rediscovering the purpose of the debriefing phases	The way of asking questions matters
	Stop the analysis when the participant began to refer to self-criticism without reinforcing positives		Be more aware that I must allow space for emotion and avoid analyzing prematurely
Constructivist learning	She relates debriefing to clinical practice: just as with patients, you cannot force change, only invite it	Learner-centered reflection	Practice closing the debriefing as one would practice making closure commitments in a therapeutic alliance
	Incorporates clinical experience to show how students learn from reflection		Help students independently identify what they learned and how they will apply it in the future
Cultural anchoring through metaphor	Lead to reflection with simple examples, like the amusement park and the balloons	Emotional resonance and conceptual clarity	The importance of starting the inquiry from a positive perspective
	Uses one “The prisoner’s” song to describe going off on a tangent		Explore other forms of debriefing that allow deeper and more effective reflection
Strategic questioning	Use A&I and five whys for the instructor to explore aspects of improvement	From feedback to reflective dialogue	Reflect more during the debriefing and explore how actions affect others’ performance
	Use circular questions and close with a question about what will be used in the next debriefing		I will use targeted open-ended questions
Emotional normalization and role modeling	Normalizes gaps in the application of the debriefing structure	Instructor self-compassion and growth	Change the way the session is closed—there is no need to overprocess what they have learned
	The instructor is uncomfortable with her debriefing. The expert manages to focus the reflection on what matters		Commit to stepping out of my comfort zone

#### Structured reorientation and the rediscovery of the debriefing phase’s purpose

When debriefers deviated from the intended structure of the debriefing, rushing, skipping phases, or over-focusing on errors, the meta-debriefer gently redirected them back using reframing strategies. These included summarizing, sequencing aloud, or asking debriefers to pause and name the debriefing phase that stood out in the conversation.

These strategies shifted debriefers’ understanding of structure from a checklist to a reflective map, one that supports psychological safety and cognitive clarity. Debriefers began to realize that flow and structure are not opposing forces but rather mutually reinforcing.

#### Constructivist learning as a path to learner-centered reflection

When the meta-debriefer drew on constructivist theory, connecting debriefing to debriefers’ clinical background or emotional experiences, participants tended to shift from didactic intentions to learner-centered approaches.

These strategies reframed the role of the debriefer, shifting it from knowledge giver to learning facilitator. In a Latin American context, where healthcare teaching often mirrors hierarchical clinical models, this repositioning offered a powerful cultural shift toward horizontal, learner-driven dialogue.

#### Cultural anchoring through metaphor to foster emotional resonance and conceptual clarity

Building on the theoretically driven work above, the meta-debriefer often drew on culturally embedded metaphors to explain reflection techniques, bridging abstract theory with familiar experience. This storytelling approach helped participants recognize themselves in the learning, often triggering personal insights, shifting from constructivist labeling to constructionist co-construction of meaning.

As illustrated earlier (e.g., the tangent metaphor and the *boli* pause), by evoking shared cultural narratives, the meta-debriefer promoted understanding not just cognitively but affectively, making facilitation feedback concrete and memorable while strengthening shared understanding. Debriefers were not only thinking differently; they were *feeling* differently about their role in the learning process.

Rather than elaborating theory, these cues performed practical functions signaling structure and focus, normalizing emotion, and reducing defensiveness. In this way, metaphor operated as a structural strategy: it made rules and transitions visible, lightened cognitive load, and reinforced relational trust, effects that are especially useful in time-pressured debriefings and diverse, interprofessional teams.

#### Strategic questioning as a bridge from feedback to reflective dialogue

The meta-debriefer consistently modeled and explained the use of targeted open-ended questions, helping debriefers shift their approach from commentary to facilitated inquiry. Particular emphasis was placed on *A&I*, five whys, and circular questions that explore interdependence within the scenario.

Debriefer often recognized their overreliance on feedback or Plus-Delta and expressed eagerness to explore more dialogic, flexible structures. Importantly, many noted that they were previously unaware of how they influenced conversations during debriefing, and now they had new tools.

#### Emotional normalization and role modeling to cultivate debriefer self-compassion and growth

By affirming emotions and naming common debriefer struggles, the meta-debriefer reduced performance anxiety and modeled reflective vulnerability. These acts often led to insights around self-compassion and confidence-building.

Rather than protecting the debriefer from discomfort, the meta-debriefer walked with them through it, using tone, metaphor, and pacing to foster insight. This mirrors a broader cultural value in Latin America: “acompañamiento” or walking together, which contrasts with more directive mentoring styles.

## Discussion

This concurrent mixed-methods research, conducted in an intrinsic case study, illustrates how deliberate reflection strategies in meta-debriefings guided by an experienced debriefer, when implemented with cultural sensitivity, can foster deep learning in novice simulation debriefers [2]. The practice of meta-debriefing analyzed in this study aligns with current trends emphasizing the importance of culturally responsive facilitation and the construction of psychological safety as foundational to effective learning [3, 22]. The approach used by the debriefer demonstrates that meta-debriefing is not merely the presence of questions or structured protocols that engender insights but also how they are delivered, the meanings they evoke, and the relational context in which they are embedded [3, 22].

This case study highlights how a skilled meta-debriefer activates learning not by delivering insight but by co-constructing it through culturally resonant strategies [9]. Whether by reframing the structure, drawing on shared metaphors, modeling emotional transparency, or teaching question formulation, the meta-debriefer transformed reflection into a pedagogical encounter, one grounded in theory, adapted to the context, and rich in relational depth [10, 19].

The observed facilitation style involves blending CORE structure with flow, empathy with inquiry, and correction with storytelling, which, in other contexts, has shown positive effects on framing affective learning in students participating in nursing simulations [27]. In Latin America, where narrative, relational closeness, and emotional authenticity are highly valued, these strategies resonate strongly [28]. The use of cultural anchors allowed debriefers to personalize abstract concepts, facilitating deeper engagement and promoting autonomy over dependency, consistent with global literature emphasizing learner-centered and culturally attuned debriefing practices [29]. This aligns with recent findings by O'Shea et al. [8], who demonstrate how facilitating authentic reflection and creating a safe space are key to fostering meaningful engagement during meta-debriefings.

The consistent use of CORE by the meta-debriefer provided role modeling of structured debriefing practice, even when debriefers varied in their own approaches. According to Abuledba [5], role modeling is significant for faculty development, as it reinforces best practices and supports the progressive alignment of debriefers’ debriefing methods with evidence-based frameworks. Otherwise, mentorship and companionship are a better approach than authority for faculty development [8].

The results suggest that CORE meta-debriefers should be structured, proficient, and flexible in their practice while prioritizing psychological safety, active listening, and constructive inquiry. Consistent guidance on reflective practice, a focus on actionable outcomes, and a balance between positive and critical feedback enhance learning [9]. Emphasizing the professional and cultural context while fostering open, formative discussions allows participants to engage deeply, underscoring the need for adaptable, learner-centered debriefing models that support reflective and practical application [6].

### Impact and strengths

This approach has the potential to promote sustainable, structured, and culturally adapted reflective practices between simulation debriefers. Cultivating a faculty development environment that integrates local cultural elements and emotional resonance reflects the importance of theoretically driven and psychologically safe meta-debriefing pillars, enhancing the relevance and acceptance of high-quality simulation-based education [2, 9]. Particularly in Latin America, our results support the broader trend toward contextually tailored faculty development [10, 12].

The main strength of this study lies in its focus on culturally resonant practices and the application of the meta-debriefing framework in a Latin American setting, an underexplored area. Furthermore, employing a mixed-methods design offers a comprehensive view of both observable behaviors and reflective processes, reinforcing the evidence base for the effectiveness of culturally adapted debriefing strategies [19].

### Limitations

However, this study also has notable limitations. Its focus on a single debriefer and a limited number of sessions constrains the extent to which findings can be generalized to wider populations or different contexts. The small sample size and the specific cultural setting require cautious interpretation; further research is needed to examine how these reflective strategies function with less experienced debriefers across varied institutional environments.

Moreover, while the qualitative insights emphasize cultural and relational dynamics, quantitative instruments like DASH and SET-M offer only a partial lens into the multifaceted skills involved in reflective facilitation, often requiring more nuanced, context-specific evaluation tools [21, 22]. Our coding scheme privileges observable structure (e.g., phase enactment) and may under-represent adaptive expertise (responsive departures from structure). Phase adherence should not be equated with effectiveness, and high-quality performance can occur with partial or selective enactment when justified by learner/contextual cues. Future work should incorporate measures of responsiveness/adaptation, link process indicators to downstream learning and team outcomes, and test these metrics across settings and cultures to refine their interpretive value.

### Trends and future directions

The increasing interest in meta-debriefing, as discussed in recent literature [8, 9], suggests a broader trend toward developing faculty’s reflexivity and relational skills to enhance learning outcomes. This aligns with the global movement in simulation education, shifting from the acquisition of technical skills to fostering more comprehensive facilitator competencies that support cultural and emotional connections [2, 3]. The findings underscore the importance of integrating cultural and relational elements into faculty development programs, particularly in Latin America, where relational trust and narrative are central to learning processes and can be tools to change hierarchical structures both in education and healthcare.

## Conclusion

In conclusion, this study offers valuable insights into how culturally sensitive and reflective debriefing enhances debriefer effectiveness and learner engagement. The strengths of the approach, its cultural resonance, and emphasis on relational strategies make it a promising model for sustainable faculty development. Nevertheless, further studies with larger, more diverse samples are necessary to strengthen the evidence base, address issues of generalizability, and explore the long-term impact of these strategies on debriefer growth and learner outcomes.

## Data Availability

The datasets generated and analyzed during this study are available from the corresponding author upon reasonable request.
